# Identification of Peach NAP Transcription Factor Genes and Characterization of their Expression in Vegetative and Reproductive Organs during Development and Senescence

**DOI:** 10.3389/fpls.2016.00147

**Published:** 2016-02-16

**Authors:** Fang Li, Jinjin Li, Ming Qian, Mingyu Han, Lijun Cao, Hangkong Liu, Dong Zhang, Caiping Zhao

**Affiliations:** College of Horticulture, Northwest A&F UniversityYangling, China

**Keywords:** *Prunus persica*, NAP subfamily, fruit, development, ripening

## Abstract

The NAP (NAC-like, activated by AP_3_/P_1_) transcription factor belongs to a subfamily of the NAC transcription factor family, and is believed to have an important role in regulating plant growth and development. However, there is very little information about this subfamily in Rosaceous plants. We identified seven *NAP* genes in the peach genome. *PpNAP2* was categorized in the NAP I group, and contained a conserved transcription activation region. The other *PpNAP* genes belonged to the NAP II group. The expression patterns of the *PpNAP* genes differed in various organs and developmental stages. *PpNAP1* and *PpNAP2* were highly expressed in mature and senescing flowers, but not in leaves, fruits, and flower buds. *PpNAP3* and *PpNAP5* were only expressed in leaves. The *PpNAP4* expression level was high in mature and senescing fruits, while *PpNAP6* and *PpNAP7* expression was up-regulated in mature and senescent leaves and flowers. During the fruit development period, the *PpNAP4* and *PpNAP6* expression levels rapidly increased during the S1 and S4 stages, which suggests these genes are involved in the first exponential growth phase and fruit ripening. During the fruit ripening and softening period, the *PpNAP1*, *PpNAP4*, and *PpNAP6* expression levels were high during the early storage period, which was accompanied by a rapid increase in ethylene production. *PpNAP1*, *PpNAP4*, and *PpNAP6* expression slowly increased during the middle or late storage periods, and peaked at the end of the storage period. Additionally, abscisic acid (ABA)-treated fruits were softer and produced more ethylene than the controls. Furthermore, the *PpNAP1*, *PpNAP4*, and *PpNAP6* expression levels were higher in ABA-treated fruits. These results suggest that *PpNAP1*, *PpNAP4*, and *PpNAP6* are responsive to ABA and may regulate peach fruit ripening.

## Introduction

The development and maturation of plant tissues involve complex processes regulated by genetic, hormonal, and environmental factors (Wang, 2008). The NAP transcription factor is a member of a subfamily of the plant-specific NAC (NAM, ATAF1, 2.CUC2) transcription factor family, which is important in many vital biological processes during plant growth and development (Sablowski and Meyerowitz, 1998; Fernandez et al., 2006; Fan et al., 2015). Sablowski and Meyerowitz (1998) determined that AtNAP is associated with cell expansion in specific *Arabidopsis* *thaliana* flower organs, while Guo and Gan (2006) reported that AtNAP is important for leaf senescence. This was further supported by a study that revealed AtNAP regulates leaf senescence processes by directly binding to the promoter of *SAG113* to form an ABA-AtNAP-SAG113 PP2C regulatory chain that controls stomatal movement and water loss in senescing leaves (Zhang and Gan, 2012). Other studies have demonstrated that NAP affects leaf senescence in bamboo (Chen et al., 2011), crocus (Kalivas et al., 2010), *Festuca arundinacea* (Guo et al., 2010), *Asarina procumbens* (Fan and Zhao, 2014), and rice (Ooka et al., 2003). Kou et al. (2012) reported that *AtNAP* expression increased during silique senescence in *A. thaliana*. Fernandez et al. (2006) observed that *VvNAP* may be important for grapevine flower and fruit development. In *Citrus sinensis* (L.) Osbeck, *CitNAC* expression was detected only in the fruit peel and pulp during the fruit ripening or senescence stages (Liu et al., 2009). Additionally, recent studies showed that the NAP subfamily is also important for regulating plant senescence and response to abiotic stresses (Meng et al., 2009; Zhang and Gan, 2012; Huang et al., 2013). The NAP transcription factor has been identified in various plant species, including rice (Ooka et al., 2003), bamboo (Chen et al., 2011), wheat (Cristobal et al., 2006), cotton (Meng et al., 2009), grape (Fernandez et al., 2006), maize (Fan et al., 2014), and soybean (Meng et al., 2007). However, the effect of NAP on the development of Rosaceae plants has not been studied.

Peach (*Prunus persica)* is an economically important crop, whose typical climacteric fruit undergoes a program of enhanced ethylene production and an associated increase in respiration rate at the onset of ripening (Barry and Giovannoni, 2007). Therefore, peach fruit softening and senescence rapidly occur after harvest, which makes storage and transport difficult. This limits peach production. A more thorough characterization of the physiological basis of peach fruit growth and ripening will enable the development of effective strategies to regulate these processes. Furthermore, peach, as a stone fruit, exhibits a typical double sigmoid growth pattern during fruit development, with distinct growth stages (S1–S4). The S1 stage corresponds to the first exponential growth phase, and is characterized by a rapid increase in cell division and elongation. In the S2 stage, which proceeds more slowly than S1, most of the dry matter is involved in pit hardening and seed and embryo growth. The S3 stage represents the second exponential growth phase, during which the fruit rapidly increases in size. Fruit ripening occurs in the final stage (S4) (Li et al., 1989; Tonutti et al., 1997; Soto et al., 2013).

In this study, we identified seven members of the peach NAP subfamily and analyzed their expression during leaf, flower, and fruit development and senescence. We revealed that members of this subfamily may function in the development and maturation of flowers and fruits, and regulate fruit softening.

## Materials and Methods

### Plant Materials

Peach tree (*P. persica* cv. ‘Qinguang 8’) samples were collected from the Experimental Station of the College of Horticulture at the Northwest A & F University in Yangling, Shaanxi, China. Samples included flowers, leaves, and fruits. Flower samples consisted of flower buds, blooming flowers, and flowers 2 days after full bloom. Young leaves were those that had just unfolded, and were collected from new shoots, while mature and senescing leaves were collected from the middle sections of new shoots. Young, mature, and senescing fruits were collected 42, 107, and 131 days after full bloom (DAFB), respectively. For fruit development analyses, young fruits were hand-picked 25 DAFB, and samples were collected every 15 days until the fruits reached commercial maturity (i.e., fruits with light green or partially red peels and slightly hard flesh). At least 20 fruits at each developmental stage were used to determine fruit weight, diameter, and gene expression.

For storage analyses, fruits with no visible defects were randomly hand picked at commercial maturity and divided into two groups. One group was soaked with 100 mM abscisic acid (ABA) for 10 min at 25 ± 1°C. The other group was soaked with water and served as the control group. Each group consisted of 120 fruits, which were kept in individual plastic bags at 25 ± 1°C. During the storage period, fruit samples were collected every 2 days, until the flesh fully softened. All samples were frozen with liquid nitrogen and stored at -80°C.

### RNA Extraction and Reverse Transcription

Total RNA was extracted using cetyltrimethylammonium bromide (Chang et al., 1993), and reverse transcription was completed using the PrimeScript RT Reagent Kit with gDNA Eraser (Takara).

### Identification of Peach NAP Subfamily Members

*Arabidopsis thaliana*, *Vitis vinifera*, and *Solanum lycopersicum NAP* gene sequences were used to search the peach genome database^1^ with the NCBI BLASTp tool to identify peach genes that were highly homologous to NAP subfamily genes.

### Multiple Sequence Alignment, Phylogenetic Analysis, and Exon/Intron Structure Determination

The NCBI BLAST tool^2^ was used to assess sequence similarities. The open reading frames of *PpNAP* genes were analyzed using the NCBI Open Reading Frame Finder tool^3^ Multiple sequence alignment analyses were conducted using the DNAMAN program, and graphical annotations of consensus sequences were completed using the Weblogo online tool^4^ A phylogenetic tree was generated using the NJ method (with 1,000 repeats) of the MEGA 6.06 software. Genetic structure investigations were conducted using the Gene Structure Display Server online tool^5^ Signal peptides were analyzed with the SignalP program^6^ (version 3.0; Bendtsen et al., 2004). Protein molecular weights and pIs were calculated using the ExPASy Compute pI/Mw tool^7^

### Molecular Cloning of Peach *NAP* Subfamily Members

To clone the *PpNAP* genes, Primer Premier 6.0 was used to design gene-specific primer pairs according to the peach genome sequence (**Table 1**). Using cDNA templates, PCR was completed with the Phanta Super-Fidelity DNA Polymerase (Vazyme) according to the manufacturer’s recommended procedure. The PCR products were isolated and purified with the MiniBEST Agarose Gel DNA Extraction Kit Ver. 4.0 (Takara). Purified products were inserted into the pMD-19T vector (Takara). Positive clones were confirmed by blue/white plaque assays. Primers for cloning and quantitative reverse transcription (qRT)-PCR were synthesized by Sangon Biotech (Shanghai) Co., Ltd, which also completed all DNA sequencing reactions.

**Table 1:** Peach *NAP* genes identified in this study.

Gene name	Gene locus	Chromosome no.	Genbank accession no.	Deduced polypeptide			Signal
				Length (aa)	MW(kDa)	PI	peptide
*PpNAPl*	*ppa007445m*	7	EMJ03289	383	42.91	8.23	–
*PpNAP2*	*ppa009530m*	1	EMJ 24523	288	33.19	7.01	–
*PpNAP3*	*ppa020620m*	4	EMJ15685	385	44.51	6.37	–
*PpNAP4*	*ppa007577m*	4	EMJ 12674	363	40.35	7.78	–
*PpNAP5*	*ppa017586m*	6	EMJ 09136	348	39.83	8.18	–
*PpNAP6*	*ppa007314m*	4	EMJ 12652	373	41.07	8.45	–
*PpNAP7*	*ppa015363m*	6	EMJ07423	356	40.46	8.25	–

*Gene locus corresponds to annotation ID from peach (Prunus persica) genome data*.

### Quantitative Reverse Transcription PCR Assays

The qRT-PCR was conducted using the iQ5 real-time PCR system (Bio-Rad). The gene-specific primers (**Table 1**) were designed using the Beacon Designer 8.0 software (Premier Biosoft International). Each primer pair (Tm 60°C) was designed to amplify an approximately 200-bp fragment. For each sample, 1 μL cDNA, 1 μL each primer, 2 μL double-distilled water, and 5 μL 2x SYBR Premix ExTaq II (Takara) were used in a total volume of 10 μL. The two-step RT-PCR was completed using the manufacturer’s recommended program, but the annealing temperature was changed to 60°C. Samples were heated at 95°C for 10 s, cooled to 65°C for 15 s, and finally heated to 95°C at a rate of 0.1°C s^–1^ for melting curve analyses. The specific transcript accumulation was analyzed using the 2^–ΔΔCT^ method (Livak and Schmittgen, 2001). Peach 18S ribosomal RNA was used to normalize data. The amplification, melt curve and melt park of 18s ribosomal gene in all samples can be seen in **Supplementary Figure S1.** Each sample was analyzed in triplicate.

### Flesh Firmness and Ethylene Production

Flesh firmness of five randomly selected fruits was measured using the GY-4 firmness meter equipped with a 8-mm diameter probe. A small epicarp segment was peeled from two places of each fruit to enable probe attachment. Three biological replicates were measured. Ethylene production was determined as described by Liguori et al. (2004) using the Trace GC Ultra gas chromatograph (Thermo Fisher Scientific). The oven, injector, and detector temperatures were 90, 110, and 140°C, respectively.

### Search for *Cis*-Acting Elements in the Promoters of Peach *NAP* Genes

Upstream regions (2000 bp upstream of the transcription start site) of selected peach *NAP* genes were used to search the PlantCARE database for putative *cis*-acting elements (Lescot et al., 2002).

### Statistical Analyses

Gene expression levels were subjected to analysis of variance using SAS. Values are provided as the mean ± standard error (*n* = 3). The overall least significant difference (*p* < 0.05) was calculated and used to separate means.

## Results

### Identification of Peach *NAP* Subfamily Members

**FIGURE 1:**
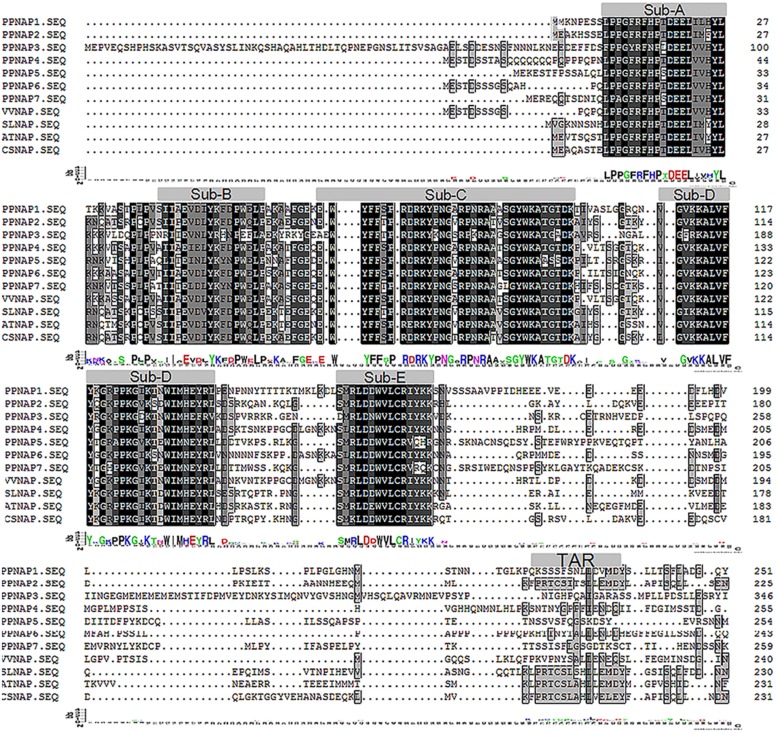
**Multiple sequence alignments of PpNAP proteins and NAP proteins from other plants.** The accession numbers of the proteins homologous to AtNAP are provided in Supplementary Table S2.

**FIGURE 2:**
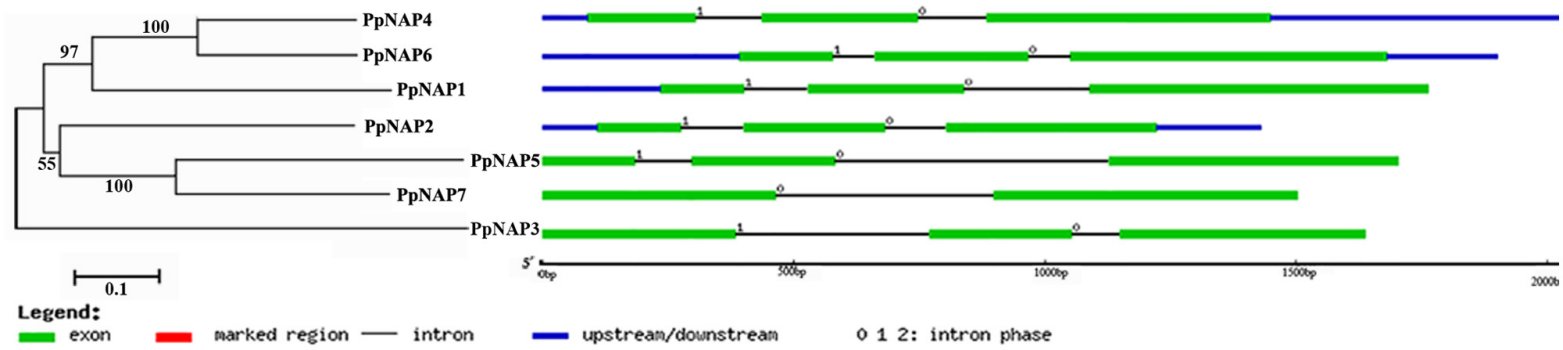
**The structures of *PpNAP* genes.** The numbers 0, 1, and 2 represent introns in phases 0, 1, and 2, respectively.

Seven *NAP* genes were detected in the peach genome with query IDs of ppa007445m, ppa009530m, ppa020620m, ppa007577m, ppa017586m, ppa007314m, and ppa015363m, which corresponded to *PpNAP1*, *PpNAP2*, *PpNAP3*, *PpNAP4*, *PpNAP5*, *PpNAP6*, and *PpNAP7*, respectively. These peach *NAP* genes contain a conserved NAC domain structure at the N-terminus, and the domain can be divided into A, B, C, D, and E subdomains. The conserved amino acid sequences in the A, B C, D, and E subdomains were LPPGFRFHPTDEELIVHYL, IIAEVDIYKFDPWELP, EWYFFSPRDRKYPNGARP NRAAVSGYWKATGTDK, VGVKKALVFYKGRPPKGYKT-DWIMHEYRL, and SMRLDDWVLCRIYKK, respectively (**Figure 1**). Furthermore, according to Fan et al. (2015), the *NAP* subfamily could be divided into two groups (NAP I and NAP II). Because of the presence of the relatively conserved transcription activation region, *PpNAP2* was included in the NAP I group, while the other *PpNAP* genes were included in the NAP II group (**Figure 1**). The *PpNAP* genes were highly homologous to *NAP* genes from other species. Similar to other *NAP* genes, *PpNAP1–6* consisted of three exons and two introns, while *PpNAP7* contained two exons and one intron (**Figure 2**). The deduced polypeptide sequences ranged from 288 to 385 amino acids, with predicted molecular weights between 33.19 and 44.51 kDa. The predicted pIs of *PpNAP* genes were from 6.37 to 8.45. None of the identified peach *NAP* genes contained signal peptide sequences according to SignalP analysis (**Table 1**).

### Phylogenetic Analysis of the Peach *NAP* Subfamily Members

**FIGURE 3:**
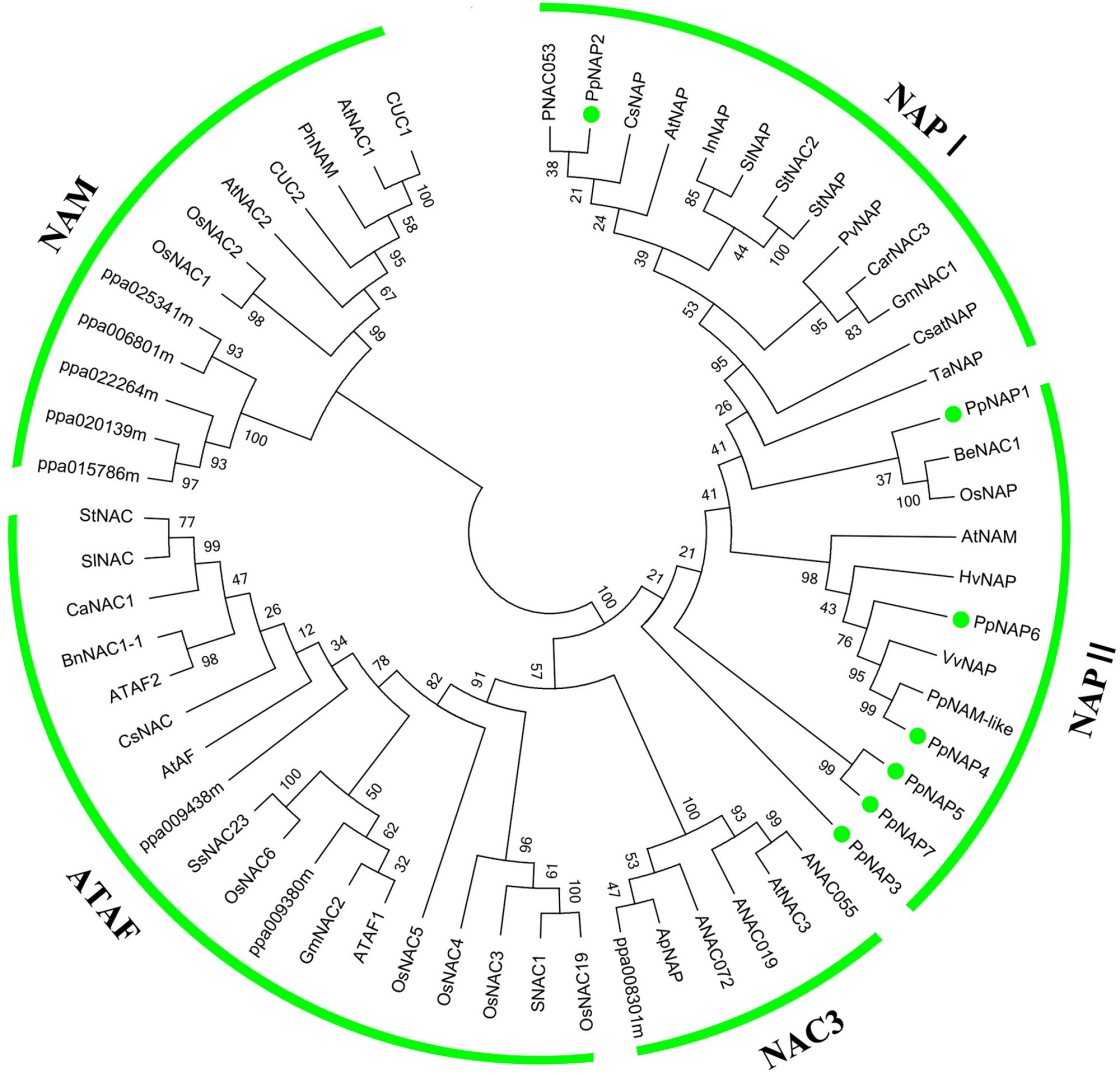
**Phylogenetic tree of *PpNAP* with NAC transcription factors from different plants.** The unrooted phylogenetic tree was constructed using the NJ method of the MEGA 6.06 program. GenBank accession numbers are provided in Supplementary Table S3.

To evaluate the evolutionary relationships among *NAP* subfamily members, cluster analyses were completed using the amino acid sequences encoded by the identified *PpNAP* genes and by *NAC* genes from potato, tomato, pepper, orange, grape, rice, *A. thaliana*, and bamboo using the MEGA 6.06 software. Phylogenetic analyses revealed that all PpNAPs are clustered in the NAP subfamily (**Figure 3**). PpNAP2 was similar to citrus, *A. thaliana*, and western balsam poplar NAPs, while PpNAP4 and PpNAP6 were similar to NAPs from grape and wheat. In contrast, PpNAP3, PpNAP5, and PpNAP7 were not particularly similar to NAPs of other plants. Additionally, the deduced amino acid sequences were more highly conserved among PpNAP4, PpNAP5, PpNAP6, and PpNAP7, while the similarities among PpNAP1, PpNAP2, and PpNAP3 were less than 28%.

### *PpNAP* Gene Expression in Various Organs at Different Developmental Stages

**FIGURE 4:**
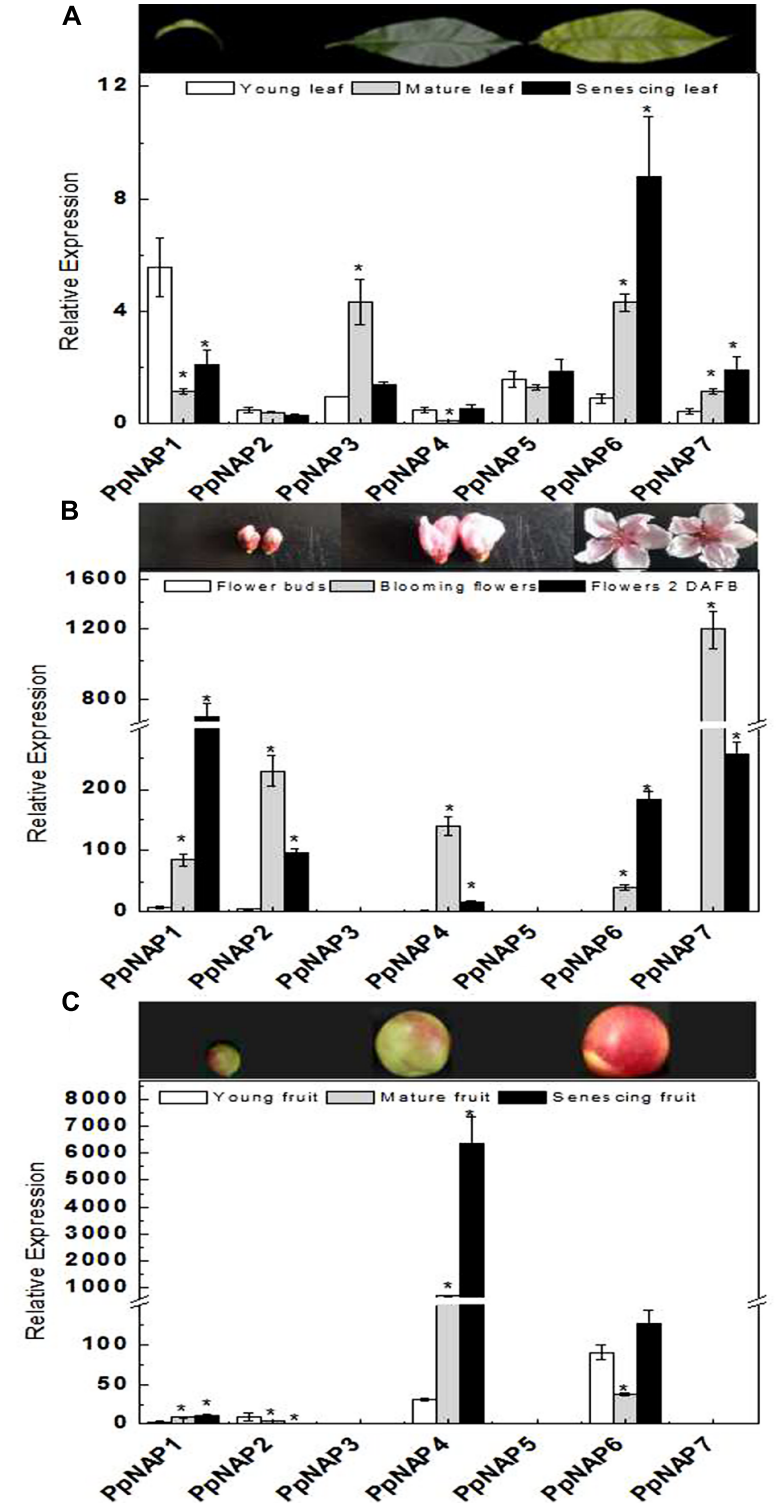
**Quantitative reverse transcription PCR analysis of selected peach *NAP* genes in leaves (A), flowers (B), and fruits (C) with different developmental stage.** The value for each sample is the mean of three replicates. Vertical bars indicate standard error. The primer sequences are provided in Supplementary Table S1. Bars correspond to the mean ± standard error (*n* = 3). Asterisks indicate significant differences in young leaves **(A)**, flower buds **(B)**, and young fruits **(C)** at *p* < 0.05 according to the Student’s *t*-test.

To investigate the potential functions of *PpNAP* genes during peach development, transcription level changes in different organs were analyzed using qRT-PCR. The *PpNAP* expression patterns were different among various organs and developmental stages (**Figure 4**). The *PpNAP* expression levels in leaves were lower than those in flowers and fruits. The expression of *PpNAP6* and *PpNAP7* rapidly increased in maturing and senescing leaves. The *PpNAP1*, *PpNAP4*, and *PpNAP5* genes were more highly expressed in young and senescent leaves than in mature leaves. In contrast, *PpNAP3* transcript levels were high in mature leaves, while *PpNAP2* expression remained relatively stable and at low levels (**Figure 4A**).

The expression levels of *PpNAP1* and *PpNAP6* were rapidly up-regulated in blooming and 2 DAFB flowers. *PpNAP2*, *PpNAP4*, and *PpNAP7* were highly expressed in blooming flowers, but expressed at very low levels in flower buds. Similarly, *PpNAP3* and *PpNAP5* expression were almost undetectable in flowers at all developmental stages (**Figure 4B**).

The *PpNAP4* and *PpNAP6* expression levels were higher than those of the other *PpNAP* genes in fruits. The higher expression levels were most obvious for *PpNAP4* in mature and senescent fruits and *PpNAP6* in young and senescing fruits. *PpNAP1* and *PpNAP2* were expressed at low levels, while *PpNAP3*, *PpNAP5*, and *PpNAP7* expression was barely detectable (**Figure 4C**).

### *PpNAP* Gene Expression Profiles During Fruit Development

To confirm the accuracy of the predicted cDNA sequences and further explore the biological functions of *PpNAP1*, *PpNAP2*, *PpNAP4*, and *PpNAP6* in fruit, we designed specific primer pairs using the peach genome sequence for cloning and expression analyses in mature ‘Qinguang 8’ fruits. The cDNA sequences of *PpNAP2*, *PpNAP4*, and *PpNAP6* were consistent with the corresponding genome sequences, while that of *PpNAP1* was 48 nucleotides longer than expected (see Supplementary Material). The expression of Pp-ACO1 is strictly related to the transition between the pre-climacteric and climacteric stage. We have analyzed the expression of Pp-ACO1 during developmental stage, and the result showed the obvious enhance of Pp-ACO1 expression at S4 stage (**Supplementary Figure S2**).

**FIGURE 5:**
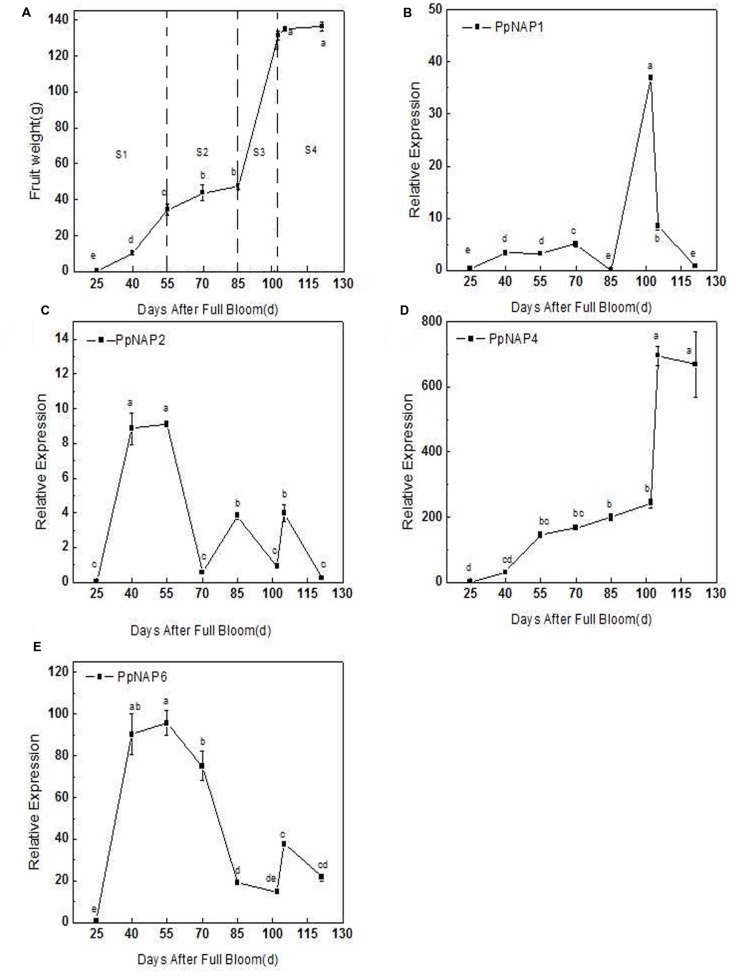
**Average fruit weight after full bloom (A), quantitative reverse transcription PCR analysis of selected peach *NAP* genes during fruit development (B–E).** Bars correspond to the mean ± standard error (*n* = 3). The overall least significant difference (*p* < 0.05) was calculated and used to separate means.

The qRT-PCR results revealed that *PpNAP4* expression in the mesocarp rapidly increased during the S1 fruit development stage (25–55 DAFB; **Figures 5A,D**), increased slowly during S2 and S3 (55–102 DAFB; **Figures 5A,D**), and significantly increased during S4, where it was maintained at a high level (102–121 DAFB; **Figures 5A,D**). In contrast, *PpNAP6* expression rapidly increased during S1, but decreased in S2, remained stable during S3, and increased during S4 (**Figures 5A,E**). The expression levels of *PpNAP1* and *PpNAP2* were low during fruit development, with elevated expression levels only during S3 (85–100 DAFB) and S1 (25–55 DAFB), respectively (**Figures 5A–C**).

### *PpNAP* Gene Expression Profiles During Fruit Ripening and Softening

**FIGURE 6:**
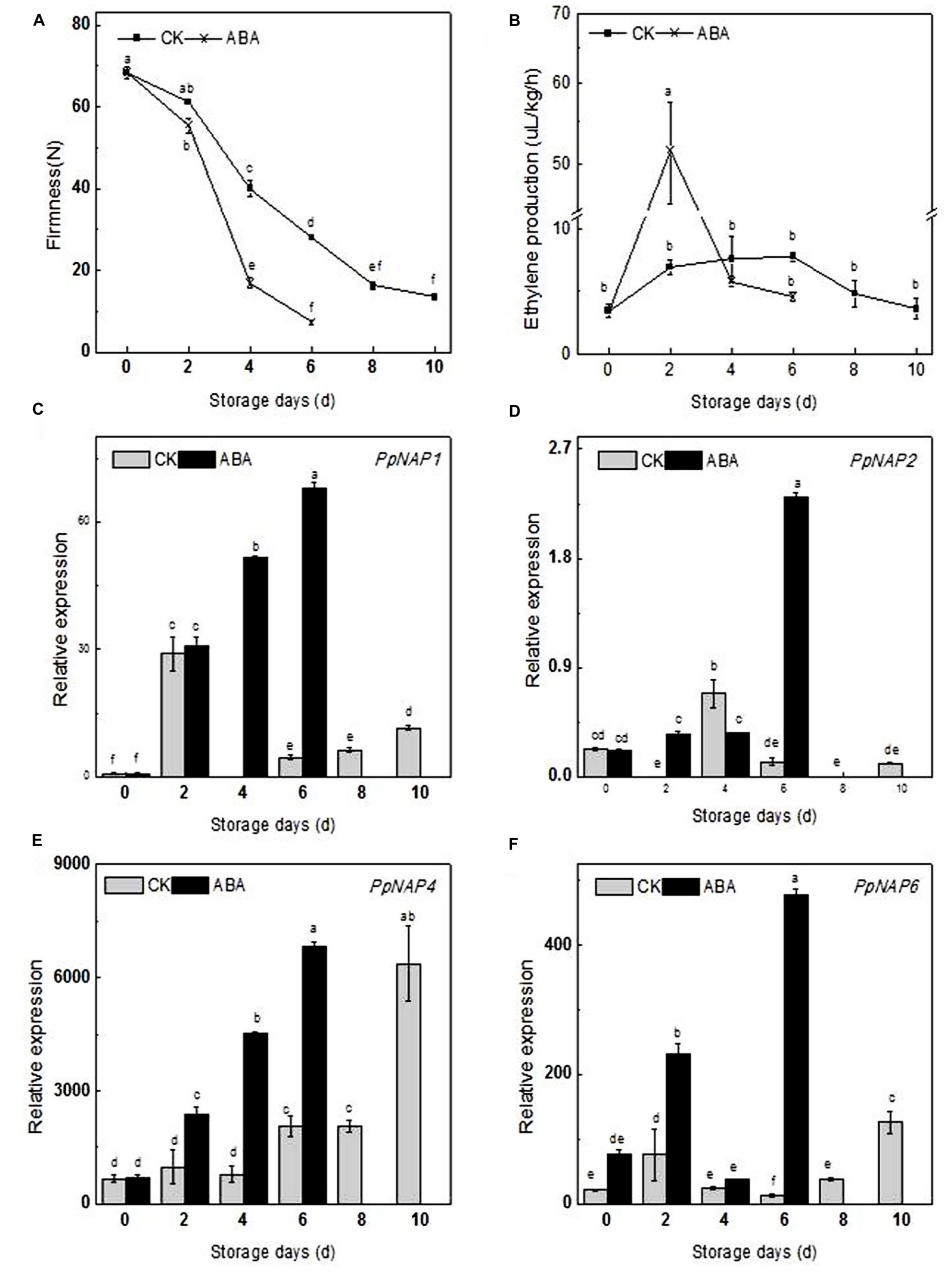
** Firmness (A), ethylene production (B) and expression levels of selected peach *NAP* genes in control and ABA-treated fruits during the storage period (C–F).** Bars correspond to the mean ± standard error (*n* = 3). The overall least significant difference (*p* < 0.05) was calculated and used to separate means.

The firmness, ethylene production, and *PpNAP* expression of commercially mature ‘Qinguang 8’ fruits were measured during fruit storage. In the first 2 days after harvest (DAH), fruit firmness decreased slowly, while from 2 to 8 DAH, fruit firmness declined rapidly (**Figure 6A**). Ethylene production doubled from 0 to 2 DAH, increased slowly from 2 to 6 DAH, and then decreased considerably (**Figure 6B**).

During storage, the *PpNAP1*, *PpNAP4*, and *PpNAP6* expression levels exhibited similar trends. Expression increased during the early storage period and was highest at 2 DAH, which coincided with the first peak of ethylene release. The expression levels subsequently declined to varying degrees. This was followed by an increasing trend from 4 or 6 DAH to 10 DAH (**Figures 6C,E,F**). *PpNAP4* and *PpNAP6* expression levels were highest at the end of the storage period (**Figures 6E,F**). In contrast, *PpNAP2* expression was maintained at a low level throughout the storage period, with highest expression levels at 4 DAH (**Figure 6D**).

### Effects of ABA Treatment on *PpNAP* Gene Expression, Ethylene Release, and Fruit Firmness

The firmness of the ABA-treated fruits was lower than that of the control fruits in the first 2 DAH, after which the firmness of the treated and control fruits decreased significantly, with treated fruits softening faster. The maximum storage periods for treated and control fruits were 6 and 10 days, respectively (**Figure 6A**).

After ABA treatment, the release rate of endogenous ethylene sharply increased and peaked at 2 DAH, with higher peak rates for treated fruits than for controls (**Figure 6B**). The *PpNAP1*, *PpNAP4*, and *PpNAP6* expression levels increased following ABA treatment for the duration of the storage period (**Figures 6E,F**). *PpNAP2* expression increased following ABA treatment at 2 and 6 DAH (**Figure 6D**).

### Sequence Analysis of *NAP* Promoters for Fruit-Specific Expression

PlantCARE database were used to identify *cis*-acting elements in the promoter regions of four *NAP* genes specifically expressed in fruit. The detected *cis*-acting elements were categorized in the following four classes: (1) involved in the perception of plant hormones, such as ABA, ethylene, methyl jasmonate, salicylic acid, and gibberellic acid; (2) related to expression elements specific to particular tissues, such as endosperm, seed, and shoot; (3) involved in transcription activation and enhancement, such as the TATA-box, CAAT-box, and 5′ untranslated region pyrimidine-rich stretch; and (4) associated with responses to environmental and physiological stimuli, such as drought, low temperature, heat stress, anaerobic conditions, light, fungal elicitors, and other stresses (**Table 2**).

**Table 2:** Details of *cis*-acting elements detected in the peach *NAP* gene promoters.

*Cis* element		Sequence	Number of *cis* elements				Function
			PpNAPl	PpNAP2	PpNAP4	PpNAP6	
Associated with plant hormones	ABRE	CACGTG	3	8	1	8	*Cis*-acting element involved in the abscisic acid responsiveness
	CE3	GACGCGTGTC	1	1	0	0	*Cis*-acting element involved in ABA and VP1 responsiveness
	ERE	ATTTCAAA	1	1	0	0	Ethylene-responsive element
	CGTCA-motif	CGTO∖	0	5	1	4	*Cis*-acting regulatory element involved in the MeJA-responsiveness
	TGACG-motif	TGACG	0	1	1	4	*Cis*-acting regulatory element involved in the MeJA-responsiveness
	TCA-element	TCAGAAGAGG	1	2	2	1	*Cis*-acting element involved in salicylic acid responsiveness
	GARE-motif	TCTGTTG	1	0	0	1	Gibberellin-responsive element
Tissue specificity expression elements	Skn-1_ motif	GTCAT	1	3	4	9	*Cis*-acting regulatory element required for endosperm expression
	RY-element	CATGCATG	2	0	0	0	*Cis*-acting regulatory element involved in seed-specific regulation
	as-2-box	GATAatGATG	0	0	0	2	Involved in shoot-specific expression and light responsiveness
*Cis* transcription functions components	TATA-box	TATA	62	79	49	60	Core promoter element around -30 of transcription start
	CAAT-box	CAAT	27	43	26	54	Common *cis*-acting element in promoter and enhancer regions
	5UTR Py-rich stretch	TTTCTTCTCT	2	0	0	1	*Cis*-acting element conferring high transcription levels
Related to physiological and environmental responsiveness	ARE	TGGTTT	4	3	3	1	*Cis*-acting regulatory element essential for the anaerobic induction
	LTR	CCGAAA	0	1	1	2	*Cis*-acting element involved in low-temperature responsiveness
	CArG-box	CN(A/T)_6_NG	3	2	1	1	MADS domain site
	HSE	AAAAAATTTC	3	0	0	0	*Cis*-acting element involved in heat stress responsiveness
	MBS	CAACTG	0	2	2	1	MYB binding site involved in drought-inducibility
	Circadian	CAANNNNATC	1	1	2	1	*Cis*-acting regulatory element involved in circadian control
	G-box	CACGTG	4	18	6	9	*Cis*-acting regulatory element involved in light responsiveness
	ACE	ACGTGGA	0	0	1	1	*Cis*-acting element involved in light responsiveness
	GTl-motif	GGTTAA	0	2	3	4	Light responsive element
	Box 1	TTTCAAA	3	1	0	1	Light responsive element
	Sp1	CC(G/A)CCC	1	3	4	1	Light responsive element
	Box 4	ATTAAT	3	3	1	1	Part of a conserved DNA module involved in light responsiveness
	ATC-motif	GCCAATCC	1	2	0	0	Part of a conserved DNA module involved in light responsiveness
	CATT-motif	GCATTC	1	0	0	2	Part of a light responsive element
	GAG-motif	GGAGATG	0	2	3	0	Part of a light responsive element
	GATA-motif	GATAGGA	1	2	0	1	Part of a light responsive element
	TCCC-motif	TCTCCCT	1	0	3	0	Part of a light responsive element
	MRE	AACCTAA	1	2	0	0	MYB binding site involved in light responsiveness
	TC-rich repeats	ATTTTCTTCA	2	1	0	0	*Cis*-acting element involved in defense and stress responsiveness
	Box-W1	TTGACC	0	2	0	2	Fungal elicitor responsive element

Among the identified *cis*-acting elements associated with hormone-related responses, the ABA-responsive element was present (one to eight copies) in all studied promoters, while the coupling element 3 was detected only in the *PpNAP1* and *PpNAP2* promoters. The CGTCA and TGACG *cis*-acting element motifs responsive to methyl jasmonate were detected (one to four or five copies) in all promoters except for that of *PpNAP1*, while the MADS-domain site CArG-box was present in all promoters (one to three copies).

## Discussion

### Identified *NAP* Subfamily Members and Sequence Analyses

The NAP is a transcription factor with crucial roles in many biological processes during plant growth and development (Fan et al., 2015). In this study, we identified seven *NAP* genes in the peach genome that were homologous to *NAP* genes from three other plant species. However, Fan et al. (2015) reported that there are four *NAP* genes in peach, corresponding to the *PpNAP1*, *PpNAP2*, *PpNAP4*, and *PpNAP6* genes identified in our study. We detected three more *NAP* genes, namely *PpNAP3*, *PpNAP5*, and *PpNAP7*. Multiple sequence alignments revealed that the seven peach NAP proteins contained the five typical NAC subdomains, and were very similar to other NAP proteins (**Figure 1**). Phylogenetic analyses indicated that all seven *PpNAP* genes clustered in the NAP subfamily, with only *PpNAP2* belonging to the NAP I group, while the others belonged to the NAP II group. This suggests the function of *PpNAP2* may differ from that of the other members.

### Tissue-Specific Expression of *PpNAP* Genes

The expression levels of the identified peach *NAP* genes were measured in leaves, flowers, and fruits, as well as during maturation and senescence. The results indicated the genes had different expression patterns, which suggests they may have different roles in various physiological pathways. *PpNAP1* and *PpNAP2* had relatively high expression levels in blooming flowers and flowers 2 DAFB, but low levels in leaves, fruits, and flower buds (**Figures 4A–C**). Therefore, these genes may be involved in regulating flower maturation and aging. *PpNAP3* and *PpNAP5* expression was observed in leaves, but was almost undetectable in flowers and fruits (**Figures 4A–C**), which indicates they may be associated with leaf development. The expression of *PpNAP4* was rapidly up-regulated and maintained at high levels during fruit maturation and senescence (**Figure 4C**), suggesting that this gene may play a key role in regulating peach fruit ripening and softening. *PpNAP6* and *PpNAP7* expression levels were up-regulated in mature and senescent leaves and flowers (**Figures 4A,B**). Therefore, they may be associated with the maturation and senescence of leaves and flowers. These results suggest that the expression of *PpNAP* genes depends on tissue type, which is supported by the results of related studies in other plants. For example, the expression of *VvNAP* was observed only in grapevine flowers and fruits, and not in vegetative organs such as leaves, shoots, or roots (Fernandez et al., 2006). The expression patterns of *AtNAP* differed among stamens, fertilized flowers, and developing siliques in *A. thaliana* (Sablowski and Meyerowitz, 1998). In *Mikania micrantha*, the *MmNAP* gene was observed to be specifically expressed in stems, petioles, shoots, and leaves, but not in roots (Li et al., 2012).

### Possible Roles of *NAP* Subfamily Members in Fruit Development and Softening

During fruit development, the *PpNAP4* and *PpNAP6* expression levels increased rapidly in stages S1 and S4. However, in the S2 stage, *PpNAP4* expression slowly increased while *PpNAP6* expression levels decreased (**Figure 5**). During the S3 stage, *PpNAP4* and *PpNAP6* expression levels stabilized. Because of the association of the *NAP* gene with cell division and expansion of stamens and petals (Sablowski and Meyerowitz, 1998), our results suggest that *PpNAP4* and *PpNAP6* are likely involved in the first exponential growth phase and fruit ripening.

During the fruit ripening and softening process, the expression of *PpNAP1*, *PpNAP4*, and *PpNAP6* increased considerably in the first 2 DAH, which was accompanied by an increase in ethylene production. Furthermore, the expression of *PpNAP1*, *PpNAP4*, and *PpNAP6* tended to increase during the middle or late storage periods, and was highest at the end of the storage period. These results are consistent with those for *AtNAP* (Kou et al., 2012), *VvNAP* (Fernandez et al., 2006), and *CitNAC* (Liu et al., 2009). Therefore, the functions of *PpNAP1*, *PpNAP4*, and *PpNAP6* are probably similar to those of *AtNAP*, *VvNAP*, and *CitNAC*, and involve activities related to peach fruit ripening and senescence.

The accumulation of ABA plays a key role in the regulation of peach fruit ripening and senescence (Zhang et al., 2009b), and stimulates ethylene biosynthesis and ripening in tomato fruits (Zhang et al., 2009a). Peach fruits treated with ABA during the S4 fruit development stage exhibited accelerated ripening and up-regulated expression of the ethylene biosynthesis genes *ACS1* and *ACO1* (Soto et al., 2013). Compared with control fruits, ABA-treated fruits softened faster and released more ethylene, ultimately resulting in a shorter maximum storage period. These results are similar to those observed for tomato (Zhang et al., 2009a), and suggest that ABA may stimulate ethylene biosynthesis. Additionally, the expression levels of *PpNAP1*, *PpNAP4*, and *PpNAP6* increased in ABA-treated fruit (**Figures 6A,B**), which was similar to the response of *AtNAP* in ABA-treated siliques (Kou et al., 2012). In rice and *A. thaliana* leaves, *NAP* gene expression was also induced by exogenous ABA (Chen et al., 2014; Yang et al., 2014). Therefore, the ABA-responsive *PpNAP1*, *PpNAP4*, and *PpNAP6* genes may regulate peach fruit ripening and softening. However, the specific regulatory mechanism requires further characterization.

### Analysis of Promoter Sequences of Selected Peach *NAP* Genes

Because of their involvement in regulating transcription, gene promoters contain important *cis*-acting elements (Zhu and Li, 1997). To characterize the possible regulatory mechanisms of *NAP* genes during fruit development, maturation, and softening, we analyzed the promoters of four fruit-specific *NAP* genes. Several motifs associated with responses to phytohormones and environmental factors were detected. These motifs included the ABA-responsive element and coupling element 3, the CGTCA and TGACG motifs associated with responses to methyl jasmonate, and the TCA-element related to responses to salicylic acid (**Table 2**). Exogenous ABA can up-regulate *NAP* expression in *A. thaliana* and rice (Liang et al., 2014; Yang et al., 2014). Zhou et al. (2013) reported that *OsNAP* can regulate leaf senescence by affecting jasmonic acid signaling pathways, and that overexpressing *OsNAP* increases the production of endogenous jasmonic acid in rice. Other studies have demonstrated that hormones, including ABA, jasmonic acid, and salicylic acid, have important regulatory roles during fruit ripening and softening (Creelman and Mullet, 1995; Zhang et al., 2003, 2009a). Therefore, it can be inferred that *PpNAP* genes regulate peach fruit development and softening by influencing specific hormone signal transduction pathways. Moreover, genes containing the MADS-box motif have key roles in flower and fruit development and maturation (Adamczyk and Fernandez, 2009; Smaczniak et al., 2012). We also observed that one to three copies of the MADS-domain site CArG-box were present in the promoters of four *NAP* genes, indicating that *PpNAP* and MADS-box genes may interact to regulate peach fruit development and ripening. However, the specific regulatory mechanisms of *PpNAP* genes that affect peach fruits require further study.

## Author Contributions

CZ, MH, HL, and DZ: Design and interpretation of all experiments. FL, JL, and MQ: Performed all plant physiological and biochemical experiments. CZ, FL and LC: Wrote the manuscript.

## Conflict of Interest Statement

The authors declare that the research was conducted in the absence of any commercial or financial relationships that could be construed as a potential conflict of interest.

## Footnotes

1 www.rosaceae.org/species/prunus_persica/genome_v1.0

2 http://www.ncbi.nlm.nih.gov/BLAST/

3 http://www.ncbi.nlm.nih.gov/gorf/gorf.html.

4 http://weblogo.berkeley.edu/logo.cgi.

5 http://gsds.cbi.pku.edu.cn.

6 http://www.cbs.dtu.dk/services/SignalP/

7 web.expasy.org/compute_pi/.

## Supplementary Material

FIGURE S1 **The amplification (A), melt curve (B) and melt park (C) of 18s ribosomal gene in all samples**.

FIGURE S2 **Quantitative reverse transcription PCR analysis of PpACO1genes in fruits with different developmental stage. Arrow indicates the time of harvest(121 DAFB)**.
