# Supplementary figures and images for: Identification of Peach NAP Transcription Factor Genes and Characterization of their Expression in Vegetative and Reproductive Organs during Development and Senescence
Source: Front Plant Sci. 2016 Feb 16;7:147. doi: 10.3389/fpls.2016.00147 (PMC4754701; doi:10.3389/fpls.2016.00147)

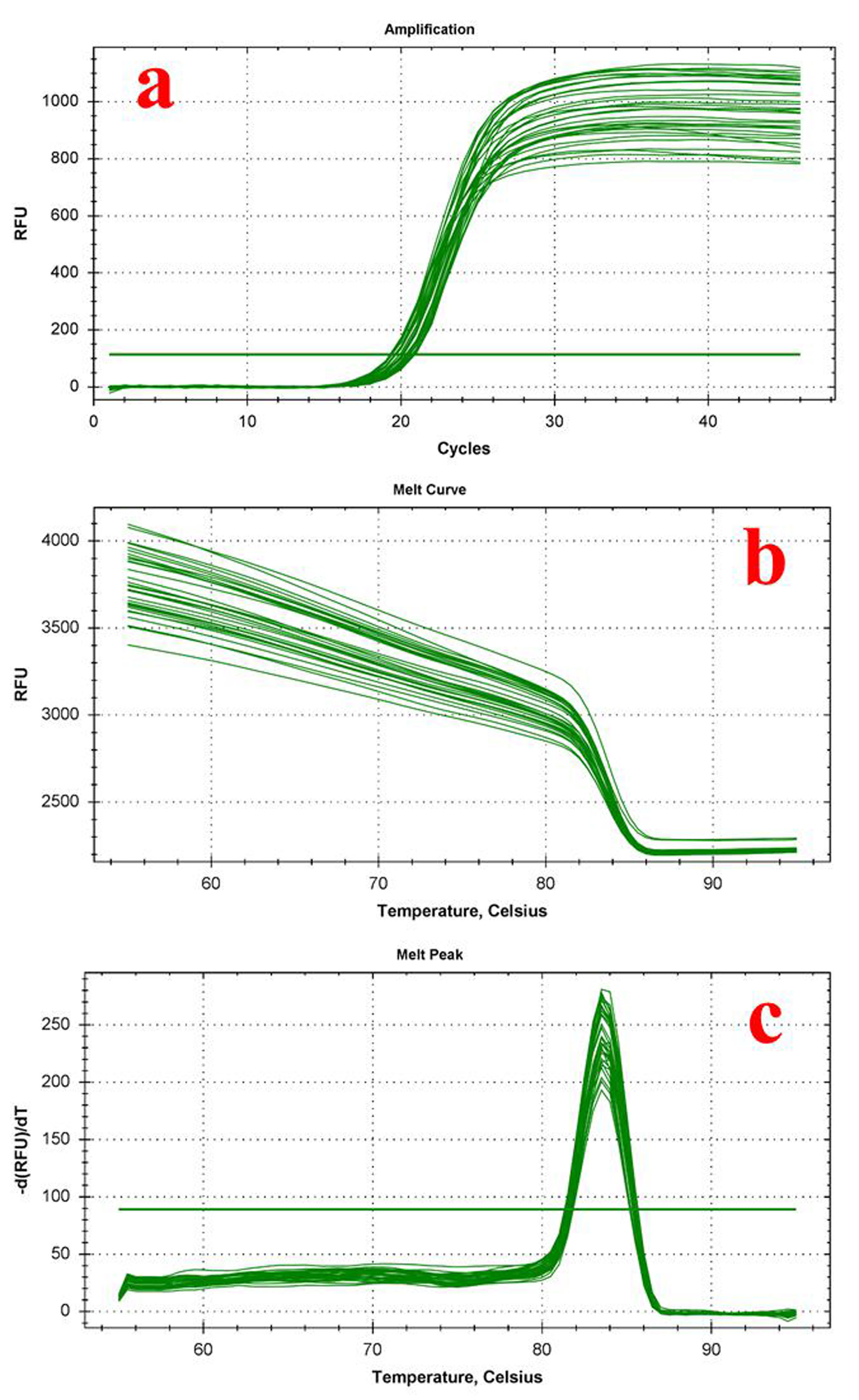

Supplement: FIGURE S1 — The amplification (A), melt curve (B) and melt park (C) of 18s ribosomal gene in all samples. [file Image_1.JPEG]

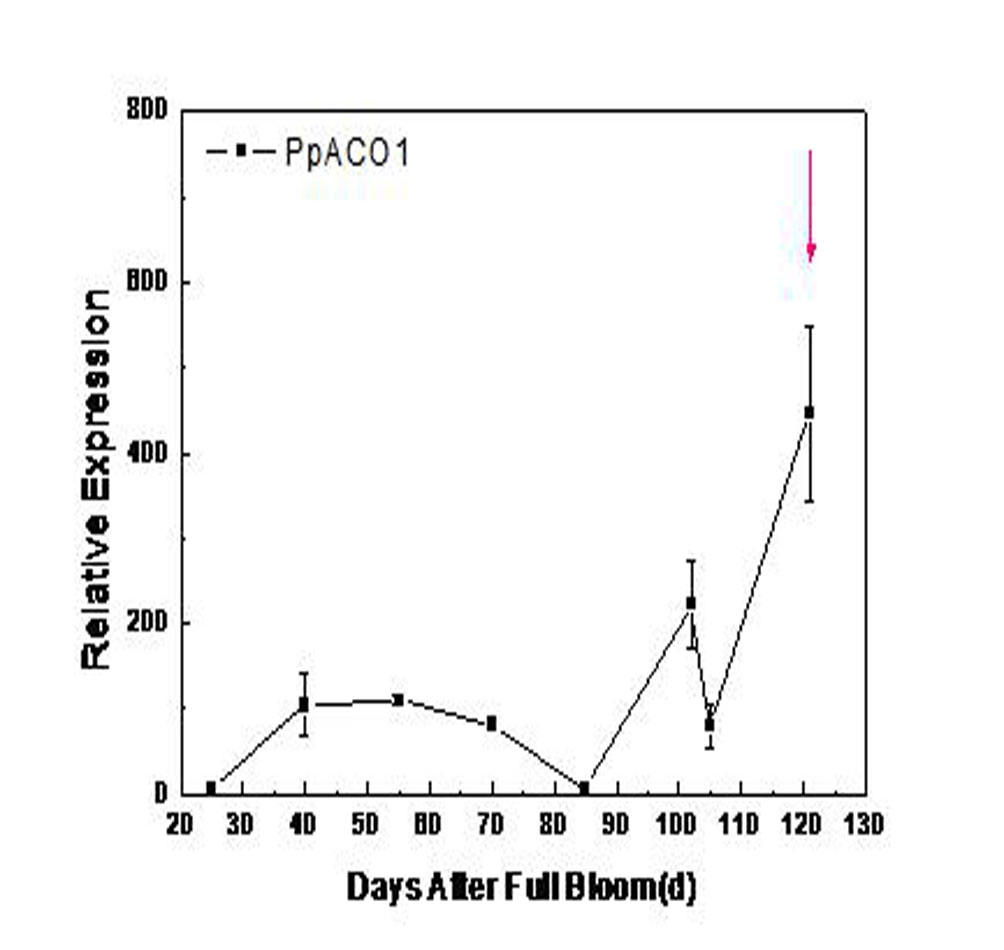

Supplement: FIGURE S2 — Quantitative reverse transcription PCR analysis of PpACO1genes in fruits with different developmental stage. Arrow indicates the time of harvest(121 DAFB). [file Image_2.JPEG]
