# Supplementary material for: Identification of Peach NAP Transcription Factor Genes and Characterization of their Expression in Vegetative and Reproductive Organs during Development and Senescence
Source: Front Plant Sci. 2016 Feb 16;7:147. doi: 10.3389/fpls.2016.00147 (PMC4754701; doi:10.3389/fpls.2016.00147)
Supplement: Supplementary file 3 [file Table_1.DOC]

Table S1

Primers and their sequences used for cloning peach NAPs and quantitative real-time PCR analysis

| Gene name | Genbank accession no. | Primers for cloning (5'-3') | Primers for qRT-PCR (5'-3') |
| --- | --- | --- | --- |
| *PpNAP1* | XM_007202028 | F:ATGATGAAAAACCCAGAATCAAGCCTG  R:TTATTCTTGAAACTGGAACTGAGGACT | F:GCTCATCCTTCACTACCTTA  R:TCGTCTTATCTGTTCCTGTT |
| *PpNAP2* | XM_007223262 | F:ATGGAGGCCAAACACAGCTCTGAG  R:TTAATTACGGTCCAAACCATTCAAGTTATAC | F:ATGAGATTGGACGATTGG  R:TGATTATTGGCTGCTGTT |
| *PpNAP3* | XM_007214424 |  | F:AACCTATACCGCCACAAT  R:CCAACAAGAGCACCATTAG |
| *PpNAP4* | XM_007211413 | F:ATGGAGAGCACCGACTCCTC  R:CTATCCCAAATTGGACTCAG | F:GTGTTGACTTCTGGAGGTA  R:ATGCGAATTGTTCTTCTTGT |
| *PpNAP5* | XM_007207875 |  | F:ATAGACTGCTTGACGATAC  R:TTCCTCTGTGTTGAACTC |
| *PpNAP6* | XM_007211391 | F:ATGGAGAGCACAGATTCATCATCGGGTTC  R:CTATGAATTCCAATTCATACTTGGGTGTTG | F:TAGTCAACAACAACAACAA  R:AATCCTCGTCCATCATAG |
| *PpNAP7* | XM_007206162 |  | F:AGATTGCTTGACACAACCAT  R:TTCTCATCCGCTTGCTTG |
