# Supplementary material for: Identification of Peach NAP Transcription Factor Genes and Characterization of their Expression in Vegetative and Reproductive Organs during Development and Senescence
Source: Front Plant Sci. 2016 Feb 16;7:147. doi: 10.3389/fpls.2016.00147 (PMC4754701; doi:10.3389/fpls.2016.00147)
Supplement: Supplementary file 4 [file Table_2.DOC]

Table S2 AtNAP homolog proteins used in Multialignment

| Species | Name | Accession |
| --- | --- | --- |
| *Arabidopsis thaliana* | ATNAP | CAA10955^a^ |
| *Bambusa emeiensis* | BeNAC1 | HM626402^a^ |
| *Crocus sativus* | CsatNAP | ABU40774^a^ |
| *Citrus sinensis* | CsNAP | ABM67699^a^ |
| *Glycine max* | GmNAC1 | AAY46121^a^ |
| *Ipomoea nil* | InNAP | AB639146^a^ |
| *Oryza sativa* | OsNAP | NP_912423^a^ |
| *Populus trichocarpa* | PNAC053 | XP_002315038^a^ |
| *Phaseolus vulgaris* | PvNAP | AAK84884^a^ |
| *Solanum lycopersicum* | SlNAP | UniGene contig 15504^b^ |
| *Solanum tuberosum* | StNAP | ABK96797^a^ |
| *Triticum aestivum* | TaNAP | AAU08785^a^ |
| *Vitis vinifera* | VvNAP | TC38700^c^ |

^a^ GenBank, ^b^ MiBASE, ^c^ TIGR plant genome database

ApNAP (*Asarina procumbens*)(Fan et al. 2014)
