# Supplementary material for: Identification of Peach NAP Transcription Factor Genes and Characterization of their Expression in Vegetative and Reproductive Organs during Development and Senescence
Source: Front Plant Sci. 2016 Feb 16;7:147. doi: 10.3389/fpls.2016.00147 (PMC4754701; doi:10.3389/fpls.2016.00147)
Supplement: Supplementary file 5 [file Table_3.DOC]

Table S3 AtNAP homolog proteins used in Phylogenetic

| Species | Name | GenBank Accession |
| --- | --- | --- |
| *Solanum tuberosum* | StNAC | CAC42087 |
| *Solanum lycopersicum* | SlNAC | AAR88435 |
| *Capsicum annuum* | CaNAC1 | AAW48094 |
| *Arabidopsis thaliana* | ATAF | NM_001297494 |
|  | ATAF1 | NP_171677 |
|  | ATAF2 | CAC35884 |
|  | AtNAM | AAD17314 |
|  | CUC1 | BAB20598 |
|  | CUC2 | BAA19529 |
|  | ANAC019 | AAT02360 |
|  | ANAC055 | AAM61076 |
|  | ANAC072 | NP_567773 |
|  | AtNAC2 | BAB20600 |
|  | AtNAC3 | BAB20599 |
|  | AtNAC1 | NP_188135 |
| *Brassica napus* | BnNAC1-1 | AAP35048 |
| *Citrus sinensis* | CsNAC | ABQ96643 |
| *Oryza sativa* | OsNAC1 | BAC53810 |
|  | OsNAC2 | BAC53811 |
|  | OsNAC3 | BAA89797 |
|  | OsNAC4 | BAA89798 |
|  | OsNAC5 | BAA89799 |
|  | OsNAC6 | BAA89800 |
|  | OsNAC19 | AAT02360 |
|  | SNAC1 | ABD52007 |
| *Glycine max* | GmNAC1 | AAY46121 |
|  | GmNAC2 | AAY46122 |
| *Saccharum officinarum* | SsNAC23 | AAW62955 |
| *Cicer arietinum* | CarNAC3 | ACO40486 |
| *Populus trichocarpa* | PNAC053 | XP_002315038 |
| *Petunia hybrida* | P.hybrida NAM | X92204 |
| *Hordeum vulgare* | HvNAM-2 | ABI94358 |
| *Prunus persica* | ppa006801m | EMJ10418 |
|  | ppa009438m | EMJ12950 |
|  | ppa015786m | EMJ12048 |
|  | ppa020139m | EMJ21331 |
|  | ppa022264m | EMJ05064 |
|  | ppa025341m | EMJ14643 |
|  | ppa008301m | EMJ12761 |
