# Supplementary material for: Identification of Peach NAP Transcription Factor Genes and Characterization of their Expression in Vegetative and Reproductive Organs during Development and Senescence
Source: Front Plant Sci. 2016 Feb 16;7:147. doi: 10.3389/fpls.2016.00147 (PMC4754701; doi:10.3389/fpls.2016.00147)
Supplement: Supplementary file 6 [file Data_Sheet_1.DOCX]

*>PpNAP1-ppa007445m(Experimental cloning result)*

*ATGATGAAAAACCCAGAATCAAGCCTGCCACCAGGGTTTAGGTTCCACCCCACAGATGAAGAGCTCATCCTTCACTACCTTACAAAGAAGGTGGCCTCCACACCCTTACCCGTTTCCATCATCGCGGAAGTTGATATCTACAAGTTTGATCCATGGGAATTACCAGCCAAAGCTGCGTTTGGTGAGAAAGAATGGTACTTCTTTAGTCCTAGAGATCGCAAGTACCCGAATGGCGCAAGGCCAAACAGGGCAGCCGCGTCAGGGTACTGGAAGGCAACAGGAACAGATAAGACGATTGTGGCGTCATTAGGAGGACGCCAGAACGTTGGTGTGAAGAAGGCTCTTGTCTTCTACAAGGGAAGACCCCCTAAAGGAATCAAGACCAACTGGATCATGCATGAATATCGCCTTCCAGAGAATCCAAACAACTACACCACCACCACCAAAACCATGAAGCTCAAAGATTTATCCATGAGGTTGGATGATTGGGTTCTCTGTCGAATCTACAAGAAGTCCAACGTCTCAAGTTCAGCAGCGGTGCCGCCAATCGATCATGAAGAAGAAGTTGAAGAAGAAGAAGATTTTCTCCATGATGTCCTGTTACCAAGCCTGAAAAGTCCCCTTCCCGGCCTTGGTCACAACATGAGTACTAATAATACTGGTCTCAAGCCCCAGAAATCTTCTTCCTTCTCCAACTTACTAGACGTCATGGACTACTCATTACTCACTAGTTTTCTGGCGGATGGCCAATACTCTAACCCAACTGGAACCGGACTCCAATCAACTCCGAATAGGTTTCCCTGTTCCGGGACTACTGGTTTAGACCAGAAACCGCTGTTCATTAATGACTATTCCAATATCAGCAGCAGCAGCAGCAGCAACAGTCATTTGCTTCAAAAGCTCCCTCAATTCAATGGTTTGAACTTGCCAGGAATCCCAAGCACCGAAAACCGGCTCAAGCGCCATGATCATCCAATTGCAGATGATCATGGTCTACTATACCCATCAAAGAAATACGTCAATTCTAATTGCAGTTTCAATAACGCAACGATTCAGTCCGACAATATCTCTCAAGGACACTTACTCAACCATTCATTGTTGAATCATCAGCAATTAGTATTGAGTAGTCCTCAGTTCCAGTTTCAAGAATAA*

There are the promoter sequences of *PpNAP1* from genomic data.

*>*promoter *PpNAP1-ppa007445m scaffold_7*

*TGAGAAGGGACGTGTAGGTCCACTGGTCTGATACCAATGATAGAACCTCGTTCAACCTTAACTAAAATGCACCGAGGGAACTATTCTTATTACTCAACAAACACAATACAATTCAACCTTTTAATACTAAAACACCCTTGGGATCCTATCACACTGATTATGTACAAAACATCAAATTAATTTTGTTCATTTCATAAATCAATCTGGTTTGAAATTTGATTTCTTTTTTCTCTTATAAATTTTGTATGCATGCAGGCTTCATAAAGAATAATAATGAGAAAGAAGAAAGAAGAAAGAAGAAGGAAGAAAAAAAGAAAGAAATTTGGTTCATAGAATGCCATCCACATCTGTATCTGACAACTTTAATATAATTTGCTTTTCAAACTTATTTGCATGATTTCCAGCCAATCCCATCAGCCCAGATGCTCCATCCATTATTTTCTTGTGTTGTCTTCTTGTGTTGTTTGAGTACTTTGCATATTGAATTGACATACTTAACATCACATTTATTATCTTCTTTTTGAAACCATCAAATGTATTATCATTGATTTTGAAGAAAAGTTATTAGTAATTTTTTTTAATCTTTATTTGTGAACATGAGTTATTATTGTTTTTGCTTCTTCTTTTGTAACATTATACAGGATTTAGAGGATGATAAATTAGAAGACTCGAGAAAGAGTGTAGAATGATAAATTAAGAGTGCGTGAAATACTTAATATAAATTTTCAAAAAATTTATGTGATCTAATGATCACTTTCAAGCTAACACCATCCCTCACGCATACGTTTGCATAAAGTAATTAAACGTATCCGGGCGATAAAAACGCCATATTAGACTATCACTATGCCCTAAACACTTACGCTGTCAAAGTTTGTATACCAAACAAACTGTAGGCTTGTACTCGTGGCAAGCGCACGCAGGGAGCGATGGGGGATAGGAATATTTCTATCTGCATTTTAAAAGTAACCTCAAAGTTCAAACGAACACTTGGAAAAATGAAAAAAAGAATAAAGTCAAGGTGAGATGCATTATTACGGCATGATGAAGATGATTGATGACTAGGCAGGCATCACAGGAGGAGCTTCTGCTTGTGAACTGTGGGGCTTCATTATTATGGGCTTGTGGACCTAGCTTTTCCAAACATGAAAGGACACGTGTCACGATTCTATCTGAGAAGAGGTTGATGATGTCACGAGAGATTAAGCTCATCCTTGGACCTTTGTCCAGCCGCCATGCATCTTCTTTATTCCATTCCATGATATCTATCTATTATTTTGTCTTCCCTCTTCTCTCTTCTCTCTTCTCCCTTCCCTTTTCGCGAAAACAAAACTTGGGAAGGACAAAACCAACAGTTCCATTCCCTTGGAGATTGGAGATTGGAGATTGGAGATTGGACCATGCATGCATGCATACATACATACGTTTCTGTTGACAAAACGATATACTGTGAGGAAAATCAAACTCGGGTGTAAGTCGACGGATGTATTATAGACATACATACATACGTATGTATAATGTATGTATATATATATAAAAGCCTTACAGCTGGCACACATACGACATCGGAGGGGAGCGTGTGTGTGGGCCTCGTATTATATTTCTTTCAGAAAATGTAACAGATGATTTGTTTTTTTCCTCAGCATCATCGGCTATTCTATTCTTCATCCCAACCCCTTGGCCTAATCTCTTCTTTTTCCGCATGCCGACAAAAAACCACAACCATAAAAGCCCTTCGTTTCTTTCTCTCCCAAAAATTTCAATTCTCGTCGTCTTTAATTTCCAGTTTGTCTTGTCACAATAGGAGTCTAGAGAGGCATAATTTCCACATCCATTTTCCTCTCTCTTACATAGAGCCAAGGCTACCTAGCTAGTTATAGCCTATCATATTACCCAACTAAACTAAACCACACAAAGCCAGCCCCTTGTATTAATTTTAAATCCTAAATCCGGTGCTCAATTAGTGCATATATAGCAAGTTTACCAGAAGGAAATATTTATTTGA*
